# Identification of Identical Transcript Changes in Liver and Whole Blood during Acetaminophen Toxicity

**DOI:** 10.3389/fgene.2012.00162

**Published:** 2012-09-04

**Authors:** Liwen Zhang, Pierre R. Bushel, Jeff Chou, Tong Zhou, Paul B. Watkins

**Affiliations:** ^1^The Hamner Institutes for Health Sciences, Research Triangle ParkNC, USA; ^2^Department of Obstetrics and Gynecology, The Fifth Hospital of Shanghai, School of Medicine, Fudan UniversityShanghai, China; ^3^Biostatistics Branch, National Institute of Environmental Health Sciences, Research Triangle ParkNC, USA; ^4^Department of Biostatistical Sciences, Wake Forest University School of MedicineWinston-Salem, NC, USA; ^5^Gentris CooperationMorrisville, NC, USA; ^6^University of North Carolina Schools of Medicine and Pharmacy, University of North CarolinaChapel Hill, NC, USA

**Keywords:** drug-induced liver injury, acetaminophen, toxicogenomics, EPIG

## Abstract

The ability to identify mechanisms underlying drug-induced liver injury (DILI) in man has been hampered by the difficulty in obtaining liver tissue from patients. It has recently been proposed that whole blood toxicogenomics may provide a non-invasive means for mechanistic studies of human DILI. However, it remains unclear to what extent changes in whole blood transcriptome mirror those in liver mechanistically linked to hepatotoxicity. To address this question, we applied the program Extracting Patterns and Identifying co-expressed Genes (EPIG) to publically available toxicogenomic data obtained from rats treated with both toxic and subtoxic doses of acetaminophen (APAP). In a training set of animals, we identified genes (760 at 6 h and 185 at 24 h post dose) with similar patterns of expression in blood and liver during APAP-induced hepatotoxicity. The pathways represented in the coordinately regulated genes largely involved mitochondrial and immune functions. The identified expression signatures were then evaluated in a separate set of animals for discernment of APAP exposure level or APAP-induced hepatotoxicity. At 6 h, the gene sets from liver and blood had equally sufficient classification of APAP exposure levels. At 24 h when toxicity was evident, the gene sets did not perform well in evaluating APAP exposure doses, but provided accurate classification of dose-independent liver injury that was evaluated by serum ALT elevation in the blood. Only 38 genes were common to both the 6 and 24-h gene sets, but these genes had the same capability as the parent gene sets to discern the exposure level and degree of liver injury. Some of the parallel transcript changes reflect pathways that are relevant to APAP hepatotoxicity, including mitochondria and immune functions. However, the extent to which these changes reflect similar mechanisms of action in both tissues remains to be determined.

## Introduction

Liver injury due to prescribed and over-the-counter medication use is a public health problem of increasing frequency and importance. Drug-induced liver injury (DILI) has been linked to nearly 1000 drugs (Abboud and Kaplowitz, 2007) and is the most common reason for regulatory actions concerning drugs, including failure of regulatory approval of new drugs, withdrawal of approved drugs from the market, and limitation in use (Watkins, 2005; Fontana, 2008). DILI accounts for more than half of the cases of acute liver failure with acetaminophen (APAP) being the principal offending drug (Lee, 2007; Tujios and Fontana, 2011).

Study of the mechanisms underlying DILI in man has been hampered by the general inability to obtain liver biopsies from patients during DILI episodes. Recent investigations have suggested that changes in whole blood transcriptome occur during acetaminophen hepatotoxicity and may precede traditional serum biomarkers in detecting and predicting this toxicity (Bushel et al., 2007; Huang et al., 2010). In one study, in healthy volunteers, a single 4 gram dose of APAP did not result in clinical or biochemical evidence of liver injury but was associated with down-regulation of genes involved in oxidative phosphorylation in whole blood (Fannin et al., 2011). These changes were interpreted as consistent with mitochondrial injury known to occur in the liver during APAP toxicity. These observations suggested that analysis of whole blood transcriptome could provide biomarkers that would be both more sensitive than those used currently to detect hepatotoxicity, and that could potentially provide insight into mechanisms that underlie hepatotoxicity. A mechanistic link between liver and blood toxicity from APAP is plausible because human and rat lymphocytes contain many of the cofactors and enzymes implicated in mechanisms of APAP hepatotoxicity, including CYP2E1, the major enzyme metabolizing APAP into its active metabolite NAPQI and glutathione, the major cofactor involved in detoxification of NAPQI (Dey et al., 2005; Hannon-Fletcher and Barnett, 2008; Dhillon et al., 2011). It has also been noted that blood lymphocyte count falls during APAP hepatotoxicity in rodents (Yamaura et al., 2002; Masson et al., 2007) which may result from cytotoxicity. However, during liver injury due to APAP, it is unclear to what extent changes in blood transcriptome mirror those occurring in liver. We reasoned that the subset of genes coordinately regulated in liver and blood during APAP hepatotoxicity would be the most useful to pursue in terms of mechanistic investigations. In addition, we reasoned that this subset of genes might be superior to other gene sets in the application of whole blood toxicogenomics to detecting hepatotoxicity from APAP.

Extracting Patterns and Identifying co-expressed Genes (EPIG; Zhou et al., 2006; Chou et al., 2007) is a transcriptome analysis program that utilizes the underlying structure of gene expression data to extract patterns and identify co-expressed genes that are responsive to experimental conditions. The program groups transcripts according to distinct patterns of induction or repression without regard to pathway or function. It is an ideal program to correlate changes in the transcriptome between two or more tissues. Gene Ontology (GO) analysis of the genes in the patterns extracted by EPIG then can identify significant categories related to biological processes (Chou et al., 2007).

We applied EPIG to publically available toxicogenomics data obtained in rats treated with toxic and subtoxic single doses of APAP (GEO Accession number GSE5652). We identified gene transcripts with similar patterns of change during APAP-induced hepatotoxicity and, in a separate validation cohort, evaluated the ability of these transcripts to discern APAP liver injury.

## Materials and Methods

### Study design

The complete transcript data set used in our analyses has been deposited at the Gene Expression Omnibus (GEO) Database under Accession number GSE5652 and whole study design can be obtained in a previously published study (Bushel et al., 2007). Briefly, Male F344/N rats were divided into training and test sets. For the training set, groups of four male rats, 12–14 weeks old, not fasted before dosing, each received 0 (vehicle only), a subtoxic dose (150 mg/kg) or two toxic doses (1500 or 2500 mg/kg) APAP in 0.5% ethyl cellulose by oral gavage. For the test set, groups of six male rats each received 0 (vehicle only), 150, or two toxic doses (1500, or 2000 mg/kg) APAP in 0.5% ethyl cellulose by oral gavage. The animals were killed at 6 or 24 h after dosing in both training and test groups. Activities of ALT were measured in all rats at study termination. mRNA was extracted from liver and blood for microarray analysis.

### Extraction of significant genes and expression signatures

Extracting Patterns and Identifying co-expressed Genes was applied as previously described (Zhou et al., 2006; Chou et al., 2007) to identify patterns among genes with significantly co-regulated expression. Briefly, the extracted intensity data from each array were preprocessed, which included array-based systematic variation normalization (Chou et al., 2005), profile-based dye swap correction, and biological reference state alignment. Through signal-to-noise ratio (SNR) evaluation applied to each correlation local cluster, EPIG extracted a set of discrete gene expression patterns. We denote each datum of log_2_ ratio as g*_ij_* in a gene expression profile, where *i* refers to a inter-group index from 1 to *m*, *j* is the intra-group index from 1 to n*_i_*, m is the number of inter-groups, and n*_i_* is the number of arrays in *i*th inter-group. To evaluate such a profile, we calculate each intra-group average ${ḡ}_{i}$ and sample variance $s_{i}^{2}$. We define a gene expression profile’s signal as

(1) $$S=\left\{-\;\begin{array}{cc} \text{max}\left\{{ḡ}_{i}\right\},\text{if min}\left\{{ḡ}_{i}\right\} & \;>\;0\\ \min\left\{{ḡ}_{i}\right\},\text{elseif}\max\left\{{ḡ}_{i}\right\} & \;<\;0\\ \text{max}\left\{{ḡ}_{i}\right\}-\text{min}\left\{{ḡ}_{i}\right\} & \;\text{otherwise} \end{array}\right.,$$

where 1 ≤ *i* ≤ *m*. We define a profile’s noise estimate as the square-root of the pooled variance, i.e.

(2) $$N=\sqrt{\frac{\overset{m}{\underset{i}{\sum }}\left[\left(n_{i}-1\right)\cdot s_{i}^{2}\right]}{\overset{m}{\underset{i}{\sum }}\left(n_{i}-1\right)}\overset{m}{\underset{i}{\sum }}\frac{1}{n_{i}}}$$

where the sample variance

(3) $$s_{i}^{2}=\frac{\overset{n_{i}}{\underset{j}{\sum }}{\left(g_{ij}-{ḡ}_{i}\right)}^{2}}{n_{i}-1}.$$

From Eqs 1 and 2, we define a profile’s SNR as

(4) $$\text{SNR}=\frac{S}{N}$$

As can be seen, when *m* = 1, Eq. 3 is equivalent to a two sample *t*-test, since by default the log2 pixel intensity ratio is the treated against its control. Equation 3 includes the case for *m* > 1, i.e., multiple inter-groups (Chou et al., 2007).

Each pattern represented a set of co-regulated genes. EPIG used the profile’s signal magnitude and SNR to identify genes with significant expression changes, and categorized them within patterns according to their correlation coefficient values.

Two-dimensional principle component analysis (PCA) and hierarchical cluster analysis (HCA) were done using PARTEK. GO categories were analyzed using Database for Annotation, Visualization and Integrated Discovery (DAVID; Huang da et al., 2009a,b). Such overrepresented categories represent biological “themes” of a given list (Hawse et al., 2005). The fact that the blood and liver tissues were from the same animals was not considered in the analysis.

### Identification of transcription factors within an expression pattern

Computational promoter analysis was done using Distant Regulatory Elements of co-regulated Genes (DiRE; Gotea and Ovcharenko, 2008). Briefly, given a target set and a background set of promoters, DiRE performs statistical tests based on a hypergeometric distribution aiming at identifying transcription factors (TFs) whose binding site signatures are significantly more enriched in the target set than in the background set. TFs were identified according to scores of two parameters: occurrence and importance. The “occurrence” represents the fraction of putative regulatory elements (REs) that contain a particular transcription binding sites (TFBS), while the “importance” is defined as the product of the TF occurrence and its weight. In the current study, co-regulated genes in each expression pattern were considered a target set and the entire set of genes represented on the microarray served as the background set.

## Results

Gene expression signatures were obtained from blood and liver of both training and test sets of animals exposed to subtoxic (150 mg/kg) and toxic (1500, 2000, or 2500 mg/kg) doses of APAP hybridized with a time-matched vehicle control (Bushel et al., 2007). Animals exposed to subtoxic dose (150 mg/kg) of APAP showed neither ALT elevation nor histopathological changes in liver at 6 and 24 h compared to vehicle treated animals. Training set animals treated with toxic doses of APAP showed significant ALT elevations (Table 1) and marked hepatocyte necrosis and degeneration (data not shown) at 24 h. ALT elevation and histopathological changes showed only minor differences among the three toxic dose groups at 24 h, so all of these animals were combined as one group for further analysis. In this study, there was a good correlation between ALT elevation and histological evidence of liver injury (Bushel et al., 2007).

**Table 1:** **ALT changes at 6 and 24 h after APAP treatment**.

Time	Training (ALT, u/L)		Test (ALT, u/L)	
	Subtoxic^a^	Toxic^b^	Subtoxic^a^	Toxic^c^
6 h	76 ± 13 (*n* = 4)	77 ± 14 (*n* = 8)	53 ± 20 (*n* = 6)	63 ± 22 (*n* = 12)
24 h	47 ± 3 (*n* = 4)	8784 ± 4948 (*n* = 8)	53 ± 7 (*n* = 6)	3648 ± 4890 (*n* = 12)

*^a^Subtoxic dose is 150 mg/kg that does not cause significant ALT elevation in comparison to vehicle only treatment; ^b^Toxic doses in training group are 1500 and 2500 mg/kg; ^c^Toxic doses in test group are 1500 and 2000 mg/kg*.

### Identification of expression signatures in the training animal set, 6 h post dose

Expression profiles from both blood and liver in the training group were analyzed for common expression patterns using EPIG at 6 and 24 h post APAP treatment using a *p*-value threshold of <0.001 for each pattern extraction. The significantly regulated genes were selected in the current study based on criteria of three parameters: correlation coefficient within a specific pattern (*r* ≥ 0.64), the magnitude of change (log2 ratio of sample vs. reference >0.4), and the SNR (SNR > 3).

**Figure 1:**
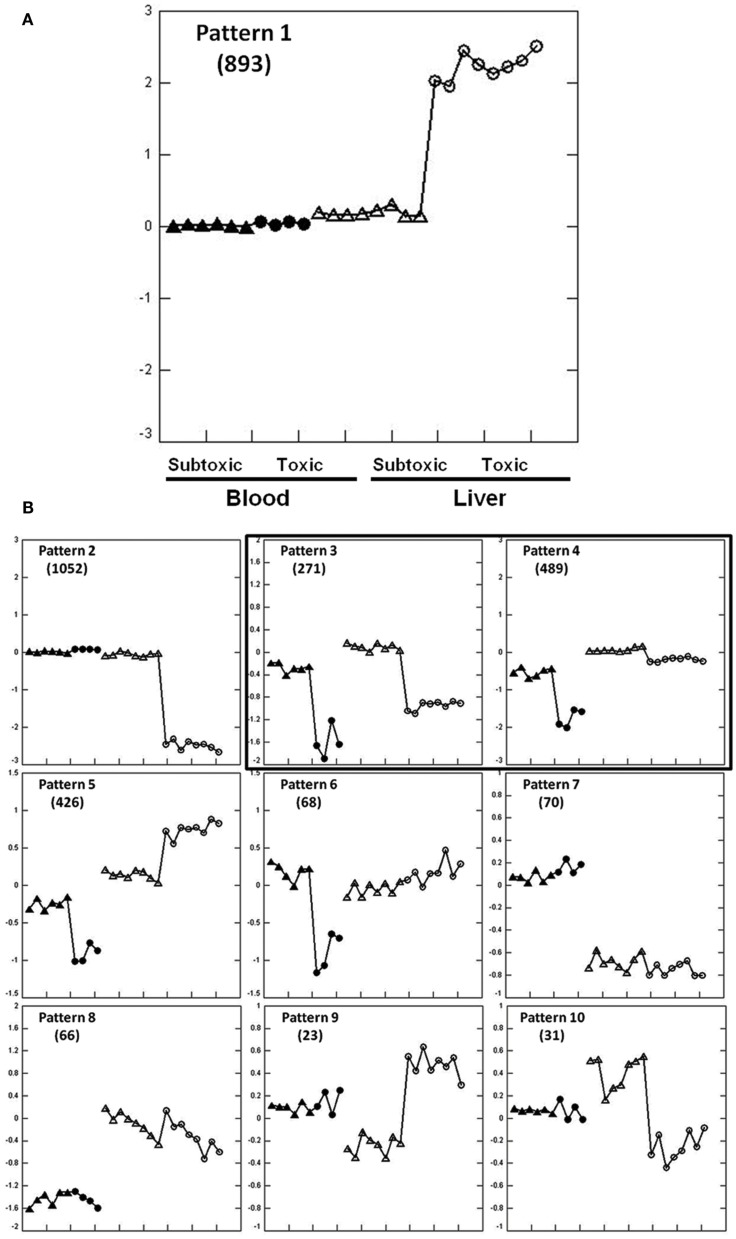
**Expression patterns at 6 h after APAP treatment extracted by EPIG for the training set animals**. Pattern 1 is enlarged to clarify the displays **(A)**. The *y*-axis is the average log base 2 of the ratio values from the top six profiles within the pattern. The ratio value is computed from the intensity measurement for each gene in each treatment sample compared to the average of the time-matched control for the sample. Zero at mid scale represents no change in gene expression, above zero means up-regulation and below zero means down-regulation. Two arrays were hybridized with Cy3 and Cy5 dye swap from one animal, so two dots represent one rat. Within a single pattern from the left to the right: blood (solid dots) and liver (empty dots); within the same tissue from left to right, subtoxic dose (triangle), and toxic doses (circle). Other gene expression patterns are shown in **(B)**. Gene numbers for each pattern are shown in parentheses. Only pattern 3 and 4 contain genes that were similarly regulated in liver and blood.

Extracting Patterns and Identifying co-expressed Genes identified 3389 genes regulated in liver and/or blood among 10 expression patterns at 6 h post dose (Figures 1A,B). The expression patterns in liver and blood are different in eight patterns (1, 2, 5–10) but similar in two patterns (3 and 4) that are of our interest in the current study. Biological pathways and functions of the genes in each pattern were analyzed using GO (Table S1 in Supplementary Material). Genes from pattern 1 and 2 accounted for most of the regulated genes (*n* = 1945) and these changes were not mirrored in liver and blood. Genes in pattern 1 were up-regulated in liver of which the most represented functions are DNA damage response and signal transduction, cell cycle arrest, negative regulation of cellular biosynthetic processes and positive regulation of transcription; genes in pattern 2 were down-regulated in liver and mainly involved in metabolic processes, including carbohydrate, steroid, and lipid metabolism and mitochondrial functions. Genes in pattern 5 and 6 were down-regulated in blood but either up-regulated (pattern 5) or showed no change (pattern 6) in liver. Functions of these genes include RNA metabolism, RNA translation and protein biosynthesis. Genes in pattern 7 and 8 showed dose-independent down-regulation in either liver (pattern 7) or blood (pattern 8) but not both. Genes in pattern 7 function in response to bacteria, lipid and cholesterol synthesis, and genes in pattern 8 largely regulate cell proliferation. Genes in pattern 9 and 10 showed no changes in blood but were changed in a dose-dependent way in liver. Genes in pattern 9 were down-regulated by the subtoxic dose but up-regulated by toxic doses. GO terms indicate they are largely related to chaperones and endoplasmic reticulum. No significant GO categories were identified from genes in pattern 10 that were up-regulated by the non-toxic dose but down-regulated by toxic dose.

There were two similarly co-regulated expression patterns, patterns 3 and 4, in liver and blood involving 760 probes that were all down-regulated in both tissues. Genes in pattern 3 (*n* = 271) were down-regulated to the same extent in both tissues and genes in pattern 4 (*n* = 489) were less down-regulated in liver than in blood. Genes in pattern 3 are principally involved in immune response, leukocyte activation, and chemotaxis. Genes in pattern 4 are largely related to mitochondrial function and oxidative phosphorylation (Tables S1 and S2 in Supplementary Material).

### Validation in the test animal set, 6 h post dose

**Figure 2:**
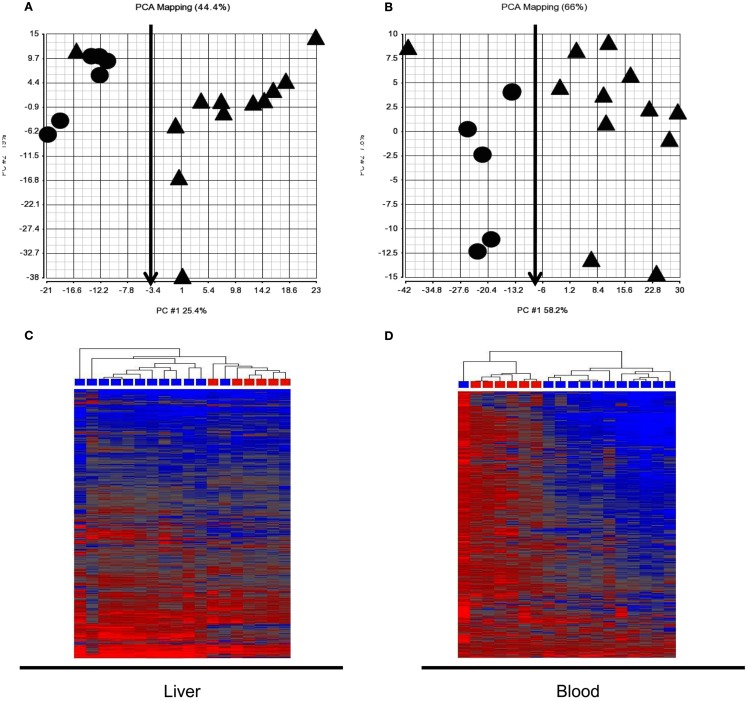
**Principle component analysis (PCA) and hierarchical cluster analysis (HCA) of the test animal set at 6 h post dose**. The 760 probes identified in the training animal as co-regulated in liver and blood at 6 h (patterns 3 and 4 in Figure 1B) reasonably distinguished the animals receiving the subtoxic dose from those receiving toxic doses. Cy3 and Cy5 array results were averaged for each rat so each symbol represents a single animal. **(A)** Liver PCA result; **(B)** Blood PCA result; **(C)** Liver HCA result; **(D)**. Blood HCA result. For HCA, samples were grouped by Euclidean distance and average linkage. Legends for **(A,B)**: circle, subtoxic; Triangle, toxic. Legends for **(C,D)** Red, subtoxic; Blue, toxic.

Expression signatures involving the 760 probes from pattern 3 and 4 identified in the training set of animals at 6 h were examined in the test animal set. PCA and HCA revealed a good separation between animals receiving subtoxic and toxic doses of APAP despite normal serum ALT at the time of sacrifice (Figure 2). However, one animal (marked with red arrows) receiving a toxic dose of APAP was segregated with those animals receiving the subtoxic dose. It is the same animal in Figures 2A,B.

### Identification of expression signatures in the training animal set, 24 h post dose

**Figure 3:**
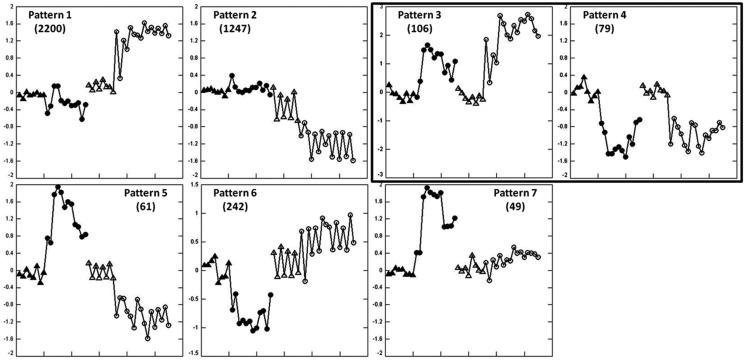
**Expression patterns in the training animal set at 24 h after APAP treatment extracted by EPIG**. The axes and symbols are identical to those in Figure 1A. Gene numbers for each pattern are shown in parentheses. Patterns 3 and 4 contained genes undergo similar regulation in liver and blood.

A total of 3984 genes were identified at 24 h as being either up or down-regulated in either liver or blood among seven expression patterns extracted by EPIG shown in Figure 3.

The regulated genes in each pattern were also subjected to GO analysis to identify enriched biological functions and pathways. Genes from pattern 1 and 2 showed significant changes in liver that were not accompanied by parallel changes in blood. These two patterns accounted for about 84% (3347) of all the regulated genes identified. Genes in pattern 1 were up-regulated in liver and are related to cell cycle regulation, cell death, and DNA repair; genes in pattern 2 were down-regulated in liver and are primarily related to oxidation and reduction, mitochondrial functions, CYPs, and metabolic processes, including steroid, cholesterol, and lipid metabolism. Pattern 5 and 6 showed changes in regulation of specific genes in both liver and blood during APAP hepatotoxicity, but the direction of regulation was opposite in the two tissues. Genes in pattern 5 were up-regulated in blood but down-regulated in liver. These genes are related to microsome and cell fraction; genes in pattern 6 were down-regulated in blood but slightly up-regulated in liver with main functions in protein catabolic processes. Genes in pattern 7 were up-regulated by toxic dose of APAP only in blood. These genes are involved in different kinds of immune processes and cell death.

A total of 185 probes were co-regulated in a similar fashion in liver and blood (patterns 3 and 4). GO analysis indicated the up-regulated genes (*n* = 106) in pattern 3 are principally related to inflammation processes and the down-regulated genes (*n* = 79) in pattern 4 play important roles in mitochondrial function (Tables S3 and S4 in Supplementary Material).

### Validation in the test animal set, 24 h post dose

**Figure 4:**
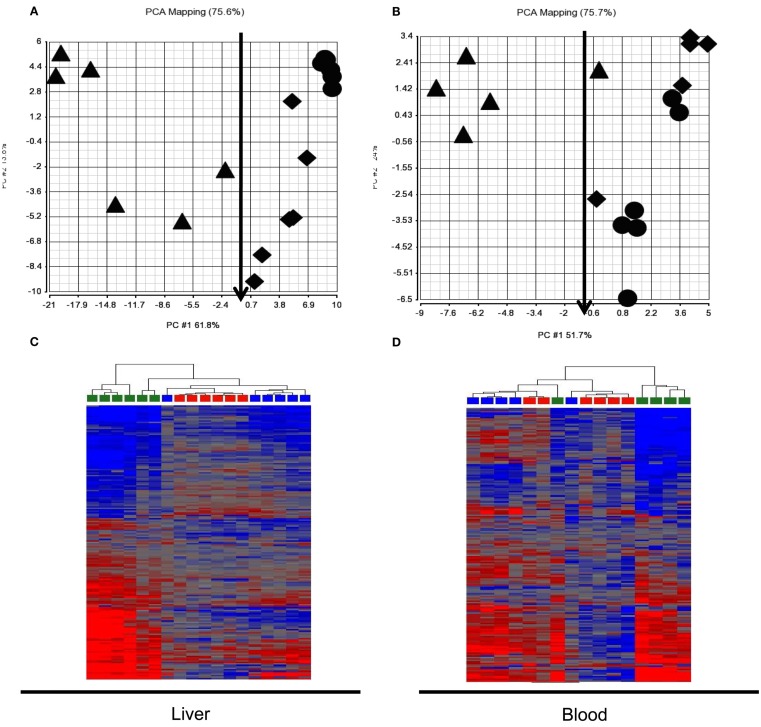
**Principle component analysis (PCA) and hierarchical cluster analysis (HCA) of test animal set at 24 h post dose**. The 185 probes identified to be co-regulated in liver and blood in the training animal set (patterns 3 and 4 in Figure 3) generally separated animals without liver injury [ALT < 100; circle and diamond in **(A,B)**; red and blue in **(C,D)**] from those with liver injury [ALT 200-12, 000; triangle in **(A,B)**; green in **(C,D)**] but had a poor classification of exposure levels, categorized as subtoxic dose [circle in **(A,B)**, red in **(A,B)**] and toxic dose [diamond and triangle in **(A,B)**, blue and green in **(C,D)**] in both liver **(A,B)** and blood **(A,B)**. **(A)** Liver PCA result; **(B)** Blood PCA result; **(C)**. Liver HCA result; **(D)**. Blood HCA result. For HCA, samples were grouped by Euclidean distance and average linkage. Legends for **(A,B)**: circle, subtoxic dose; diamond, toxic dose without liver injury; Triangle, toxic dose with liver injury. Legends for **(A,B)**: red, subtoxic dose; Blue, toxic dose without liver injury; Green, toxic dose with liver injury.

We assessed expression changes in the 185 probes from patterns 3 and 4 in the test animal set to classify APAP exposure and liver injury (Figure 4). PCA and HCA indicated that classification of exposure levels by the gene set from either liver or blood at 24 h, when toxicity was evident, was very poor when binary categories, subtoxic and toxic, were applied. About half of the animals that received toxic doses were segregated together with subtoxic dosed animals. Further analysis of ALT levels in these animals showed no elevation compared to vehicle or subtoxic dose treated animals, indicating no liver injury occurred despite administration of toxic doses of APAP. Very interestingly, the gene set, when used to classify liver injury evaluated by ALT elevation, was able to correctly separate animals with no liver injury (ALT < 100) from those with liver injury (ALT ranging from 200 to 12,000) despite the APAP exposure doses. The only one misclassified from the group with liver injury into the group without injury by blood expression signature was dosed with 1500 mg/kg of APAP and had an ALT level of 3380 U/L (marked with a red arrow). The same animal (also marked with a red arrow) is correctly classified by liver expression signature.

### Transcription factor identification for co-regulated genes upon liver injury at 6 and 24 h

Transcription factors most implicated in the co-regulated genes in liver and blood within an expression pattern (Figure 1B, patterns 3 and 4; Figure 3, patterns 3 and 4) were identified using DiRE (Gotea and Ovcharenko, 2008). The top five TFs implicated at each expression pattern at 6 and 24 h are shown in Table 2 with occurrence rates and importance scores.

**Table 2:** **Transcription factors for co-regulated genes from pattern 3 and 4 at 6 and 24 h post APAP treatment**.

Time	Pattern 3			Pattern 4		
	TF	Occurrence	Importance	TF	Occurrence	Importance
6 h	ISRE	0.1584	0.62995	FOXP3	0.0738	0.32975
	PPARA	0.1584	0.35743	ZIC2	0.0902	0.23274
	40820	0.1089	0.33558	STAT1	0.1475	0.225
	ZIC1	0.1188	0.29926	HMEF2	0.0738	0.21071
	NCX	0.099	0.20854	NRSF	0.0738	0.16368
24 h	P53	0.1111	0.27778	TBP	0.1333	0.36996
	HNF3	0.1111	0.23153	TEL2	0.1	0.26987
	HFH1	0.1111	0.23104	MYC	0.0667	0.20898
	NF1	0.1481	0.16037	SREBP1	0.1667	0.17792
	ETS1	0.1111	0.08964	STAT6	0.1333	0.16233

### Comparison of the 6 and 24 h expression profiles

We next compared the gene sets identified as having similar regulation in liver and blood at 6 or 24 h. Only 38 of the total 945 genes identified were common to both gene sets (Table S6 in Supplementary Material); GO category analysis of these 38 genes indicated enrichment of genes involved in mitochondrial function and regulation of lymphocyte and leukocyte activation (Table S5 in Supplementary Material). The expression signature consisting of only these 38 genes was able to separate the test animals receiving toxic and subtoxic APAP doses at 6 h or animals with or without liver injury at 24 h post dose, with the same performance efficiency as the parent gene sets (760 or 185) in both liver and blood.

## Discussion

Application of blood as a surrogate tissue to extract genomic indicators for cross tissue prediction of target organ injury has been reported in several studies (Bushel et al., 2007; Lobenhofer et al., 2008; Huang et al., 2010; Umbright et al., 2011). Blood transcriptomic changes have been shown to precede appearance of traditional markers of tissue injury and it has been suggested that blood toxicogenomics might provide novel biomarkers to identify patients who will develop severe liver toxicity after taking overdoses of APAP (Cui and Paules, 2010). The mechanisms underlying changes in blood transcriptome during APAP hepatotoxicity are not known. It has been proposed “that the circulating blood cells monitor the physiological state of the organism and alter their transcriptome in response to this surveillance.” (Burczynski and Dorner, 2006). Hence, the changes occurring in the blood transcriptome may reflect a reaction to hepatotoxicity of APAP. On the other hand, changes occurring in whole blood transcriptome observed during APAP toxicity in humans have been linked to known mechanisms of APAP toxicity in the liver and it has therefore been suggested that analysis of whole blood transcriptome may provide insight into mechanisms that underlie hepatotoxicity (Fannin et al., 2011). This is possible since lymphocytes contain many of the cofactors and enzymes implicated in mechanisms of APAP hepatotoxicity (Dey et al., 2005; Hannon-Fletcher and Barnett, 2008; Dhillon et al., 2011) and APAP-induced cytoxicity may account for a fall in circulating lymphocyte counts during APAP hepatotoxicity in rodents (Yamaura et al., 2002; Masson et al., 2007). The extent to which changes in liver transcriptome are mirrored in blood during APAP toxicity has not been previously reported. We addressed this question using EPIG and toxicogenomics data provided by prior rat studies of APAP-induced liver injury (Bushel et al., 2007).

We were able to successfully identify expression patterns showing similar expression changes in liver and blood in the training animal set at 6 and 24 h (Figures 1 and 3). GO analysis of biological functions of these genes within each expression patterns supported a mechanistic link to APAP hepatotoxicity. At 6 h, the genes co-regulated in liver and blood were involved in mitochondrial function, including oxidative phosphorylation (Figure 1B, pattern 4). Down-regulation of oxidative phosphorylation genes was more pronounced in blood than liver, suggesting that impairment of oxidative phosphorylation may occur earlier in blood than liver. This may reflect higher exposure to blood cells relative to liver, or increased sensitivity of specific cell types in blood. There was also coordinate down-regulation at 6 h of genes involved in immune function and inflammation (Figure 1B, pattern 3). We speculate that this may somehow represent an adaptive response prior to overt liver injury. At 24 h, biological pathways including inflammation processes and mitochondrial function were also identified among the co-regulated genes in liver and blood, showing up-regulation of inflammation processes (Figure 3, pattern 3) and down-regulation of mitochondrial function (Figure 3, pattern 4). The identified pathways at 6 and 24 h are consistent with the accepted roles of mitochondria and inflammation in APAP-induced hepatotoxicity (Lee, 2003; Jaeschke and Bajt, 2006; Larson, 2007). Although these observations are consistent with similar mechanisms of action in liver and blood, caution is appropriate in the interpretation of our data since the pathways identified could be operative by multiple mechanisms.

Application of the extracted expression signatures in a separate test set of animals yielded interesting results. Bushel et al. (2007) reported the utility of blood gene expression data to predict exposure to APAP in the same rat data set. Classifiers from multiple prediction algorithms were applied to predict the APAP exposure levels divided into nonsubtoxic and toxic without considering the duration of exposure with high accuracy (88.9–95.8%). In the current study, at 6 h when no obvious liver injury was observed, both liver and blood expression signatures had very good classification of APAP exposure levels based on a binary category: subtoxic vs. toxic (Kerns and Bushel, 2012). Huang et al. (2010) explored the use of rat blood gene expression data to predict liver necrosis. DILI was defined as a binary response (no necrosis vs. some observable sign of necrosis) without considering the dose and/or time of exposure. From the various, highly accurate predictors, 10 gene-probes occurred most often.

Changes in gene expression at 6 h may reflect an early adaptive mechanism related to exposure levels but less related to actual liver injury. Neither liver nor blood expression signature had a good APAP exposure classification at 24 h based on the same binary category. However, when liver injury measured as ALT elevation was used as endpoint for anchoring to a phenotype, both liver and blood expression signatures yielded very good classifications.

Liver injury classification by expression signatures was not able to be performed at 6 h because of lack of histological evidence and ALT elevation in all animals sacrificed at this time point. An interesting observation was one rat treated with a toxic dose of APAP co-segregated at 6 h with those animals receiving subtoxic doses of APAP (Figures 2A,B). We speculate that this animal might not have developed overt liver toxicity; since this animal was sacrificed at 6 h, there was no way to determine this. This hypothesis could be tested in a new study by assessing the transcript profile in blood at 6 h but allowing the animals to live to 24 h post dose when responder status could be determined.

In spite of similar processes and functions being identified among the co-regulated gene patterns at 6 and 24 h, there were just 38 genes present in both gene sets. Among the 38 genes, 14 genes were down-regulated at 6 h but up-regulated at 24 h, and 24 genes were down-regulated at both 6 and 24 h. We found that expression signatures using just these 38 genes had similar efficacy to the larger gene sets in distinguishing those animals receiving toxic and subtoxic doses at 6 h. In addition, this limited gene set was also capable of separating animals at 24 h based on the extent of toxicity as reflected in ALT values. Not surprisingly, 11 of the 38 genes are closely related to mitochondrial function (*Abcd3, Mccc1, Mosc2, Sdha*, and *Sdhd*) and immune response (*Ccl5, Cd1d1, Fas, Il1b*, and *Lat*).

Change in the expression of a group of genes is often the result of activation or inactivation of TFs in response to endogenous or extraneous stress. We therefore analyzed the co-regulated genes to identify the top 10 implicated TFs at 6 and 24 h. In some cases, the identified TFs may provide mechanistic insight into APAP toxicity. Activation of p53 at 24 h may suggest that DNA damage is involved in the final stages in APAP-induced liver and blood cell injury. TATA-box binding protein (TBP) plays a crucial role in transcription initiation of RNA polymerases (Muller et al., 2010; Tora and Timmers, 2010) which may stimulate protein synthesis necessary for injury repair. Interferon-stimulated response element (ISRE) and PPARα are two TFs with the highest importance score identified by DiRE at 6 h. ISRE has not been reported to be directly related to APAP hepatotoxicity. However, it plays important roles in regulation of interferon-induced gene regulation (Wesoly et al., 2007), which has been implicated in mechanisms of APAP-induced liver injury (Ishida et al., 2002; Ghaffari et al., 2011). The suppression of PPARα activation has also been reported as a contributing mechanism of APAP-induced hepatotoxicity (Chen et al., 2009).

In conclusion, similar expression changes occur in a subset of genes in blood and liver during APAP-induced liver injury and after treatment with toxic doses of APAP before onset of overt toxicity. Although the genes that demonstrated coordinate regulation between the tissues represent a small fraction of the total affected genes, the pathways, and functions linked to these genes may support the idea that transcriptomic analysis of blood can provide mechanistic insight into DILI. However, a true mechanistic link between tissues remains to be demonstrated. The ability of the transcript signatures to distinguish those receiving the identical doses but demonstrating variability in susceptibility to the toxicity supports other data suggesting that blood toxicogenomics might provide a useful avenue to predicting susceptibility to liver toxicity in the clinic.

## Conflict of Interest Statement

The authors declare that the research was conducted in the absence of any commercial or financial relationships that could be construed as a potential conflict of interest.

## Supplementary Material

The Supplementary Material for this article can be found online at http://www.frontiersin.org/Toxicogenomics_/10.3389/fgene.2012.00162/abstract
